# Trends in the prevalence of twenty health indicators among adolescents in United Arab Emirates: cross-sectional national school surveys from 2005, 2010 and 2016

**DOI:** 10.1186/s12887-020-02252-0

**Published:** 2020-07-29

**Authors:** Supa Pengpid, Karl Peltzer

**Affiliations:** 1 ASEAN Institute for Health Development, Mahidol University, Salaya, Phutthamonthon, Nakhon Pathom, Thailand; 2 Department of Research Administration and Development, University of Limpopo, Polokwane, South Africa; 3 Department of Psychology, University of the Free State, Bloemfontein, South Africa

**Keywords:** Obesity, Health indicators, Mental health violence, Protective factors, Hygiene, Injury

## Abstract

**Background:**

The aim of this study was to assess the trends in the prevalence of various health indicators among adolescents in United Arab Emirates (UAE).

**Methods:**

Nationally representative data were analysed from 24,220 in-school adolescents (median age = 14 years) that took part in three cross-sectional surveys (2005, 2010 and 2016) of the “UAE Global School-Based Student Health Survey (GSHS)”.

**Results:**

Significant improvements were identified among both girls and boys in the reduction of being physically attacked, inadequate fruit intake, inadequate vegetable consumption, loneliness, and among girls only poor oral hygiene (< 2 times tooth brushing/day) and among boys only, experiencing hunger and in physical fight. Significant rises were identified among both girls and boys in the prevalence of bullying victimization, overweight or obesity, leisure-time sedentary behaviour, injury and inconsistent washing hands prior to eating, and among boys only obesity and among girls only inadequate physical activity, and school truancy.

**Conclusions:**

Several reductions but even more increases of poor health indicators were identified over three cross-sectional surveys during a period of 11 years emphasizing the need for enhanced health promotion activities in this adolescent school population.

## Background

In United Arab Emirates (UAE), a high-income Arab country, 77% of all death are attributed to non-communicable diseases (NCDs) [1]. The prevalence of NCDs (diabetes, cancer, chronic lung diseases and cardiovascular disease) is on the rise in countries of the Arab region, including the UAE [2]. Behavioural NCD health risk indicators, such as physical inactivity, unhealthy diets, tobacco use, and obesity, are very common among children and adults in the Arab region [2]. As stated by the World Health Organization (WHO), “alcohol use, dietary behaviours, drug use, hygiene, mental health, physical activity, protective factors, sexual behaviours, tobacco use, violence and unintentional injury” are the leading causes of morbidity/mortality among children and adults globally [3]. Monitoring various health indicators, such as nutrition and diet, substance use, physical activity, violence, injury and mental health, among adolescents over time may facilitate targeting intervention strategies [4–6].

Diverse results were found in research investigating trends in health indicators among adolescents [5, 6]. For example, in a trend study among adolescents in the Philippines [5] poor hand hygiene behaviour decreased over time, while it increased in Oman [6], and interpersonal violence, injury and physical inactivity decreased, while the prevalence of fruit and vegetable intake (one study) increased [5, 6]. In terms of injury and interpersonal violence, in a large study among adolescents in the UAE, 18% reported a physical injury in the past 12 months [7]. In a local study among adolescents in UAE, 15.4% of males and 8.0% of females reported physical violence (having been hit and pushed) in the past month [8]. In another study among 1054 school students in Dubai, peer violence (beating 39.4% and boxing 24.5%) was commonly reported [9].

Regarding overweight and obesity, in a study among 6–19 year-old students in Abu Dhabi, UAE, 14.7% were measured to have overweight and 18.9% obesity [10]. In a study among adolescents in public and private schools in Dubai, 72% reported inadequate fruit and vegetable intake [11]. In a meta-analytic review of physical activity among adolescents in the UAE, one in four had total sedentary behaviour with no physical activity [12]. In a cross-sectional study (2007–2009) among adolescents in UAE, the prevalence of current smokers was 14.0% [13]. In terms of mental health, in a sample of school adolescents (N = 600) in the UAE, 17.2% were found to have depressive symptoms [14], and in another adolescent school sample (N = 968) in UAE, the prevalence of anxiety disorders was 28% [15].

There is a major research gap in the assessment of trends in health indicators over time among adolescents in the Eastern Mediterranean region, such as in UAE. The present study aims to estimate trends of the prevalence of 20 different health and five protective indicators in the 2005, 2010 and 2016 UAE “Global School-based Student Health Survey (GSHS)”. It is hypothesized that the prevalence of health indicators differs across the three GSHS from 2005, 2010 and 2016. Research results on trends of various health indicators may be beneficial for health promotion activities in schools [16].

## Methods

### Participants and procedure

Data from the 2005, 2010 and 2016 UAE cross-sectional GSHS were analysed [3]. A sampling design in two stages (first: schools selected with probability proportional to sample size, and second: classes of grades 8, 9, and 10 students within schools were randomly selected) was used to generate a national representative country sample [3]. All students in the selected classes were eligible to participate regardless of their age, and responded to a self-administered questionnaire [3]. For the 2005 UAE GSHS the response rate was 89%, for 2010 91% and for the 2016 UAE GSHS 80% [3]. The data and more detailed information on the study procedures can be accessed [3].

The GSHS core questionnaire assesses 10 modules: “alcohol use, dietary behaviours, drug use, hygiene, mental health, physical activity, protective factors, sexual behaviours that contribute to HIV infection, other sexually-transmitted infections, and unintended pregnancy, tobacco use, violence and unintentional injury.” [3] All core modules of the questionnaire that were implemented in the 2005, 2010 and 2016 UAE GSHS were part of this analysis.

### Measures

The questionnaire used is shown in Table 1 [3]. Overweight/obesity was classified as “more than + 1 standard deviation (SD) and obesity more than + 2 SD from the median body mass index by age and sex,” using the 2007 WHO Child Growth reference [17]. The consumption of less than “two or more servings of fruits in a day” and less than “three or more servings of vegetables a day” were considered inadequate [18]. “Inadequate physical activity was defined as not daily at least 60 minutes of moderate to vigorous-intensity physical activity.” [19] “Leisure-time sedentary behaviour was defined as spending three or more hours per day sitting.” [20].

**Table 1:** Variable description

Variables	Question	Response options (coding scheme)
Age	“How old are you?”	“11 years old or younger to 16 or 18 years old or older”
Sex	“What is your sex?”	“Male, Female”
**Body weight and dietary behaviour**		
Body weight	“How much do you weigh without your shoes on?”	kg
Height	“How tall are you without your shoes on?”	cm
Fruit intake	“During the past 30 days, how many times per day did you usually eat fruit such as apples, bananas, and oranges?”	“1=I did not eat fruit during the past 30 days to 7=5 or more times per day (coded 1-3=1 and 4-8=0)”
Vegetable intake	“During the past 30 days, how many times per day did you usually eat vegetables, such as salads, spinach, eggplant, tomatoes, and cucumbers?”	“I did not eat vegetables during the past 30 days to 7=5 or more times per day (coded 1-4=1 and 5-8=0”
**Physical activity and sedentary behaviour**		
Physical activity	“Physical activity is any activity that increases your heart rate and makes you get out of breath some of the time. Physical activity can be done in sports, playing with friends, or walking to school. Some examples of physical activity are running, fast walking, biking, dancing, football, swimming, and aerobics. During the past 7 days, on how many days were you physically active for a total of at least 60 minutes per day?”	“0=0 days to 7=7 days (coded 0-6=0 and 7=1)”
Leisure-time sedentary behaviour	“How much time do you spend during a typical or usual day sitting and watching television, playing computer games, talking with friends, or doing other sitting activities, such as studying or using any electronic devices like IPads?”	“1=less than 1 hour per day; 2=1-2 hrs/day; 3=3-4 hrs/day; 4=5-6 hrs/day; 5=7-8 hrs/day and 6=8 or more hours per day”
**Tobacco use**		
Current tobacco use	“During the past 30 days, on how many days did you smoke cigarettes/use any tobacco products other than cigarettes, such as Sheesha, Medwakh, chewed tobacco, or electronic cigarettes?”	“1=0 days to 7=All 30 days (coded 1=0 and 2-7=1)”
**Injury and violence**		
Injury	“During the past 12 months, how many times were you seriously injured?”	“1=0 times to 8=12 or more times (coded 1=0 and 2–8=1)”
Bullying victimization	“During the past 30 days, on how many days were you bullied?”	“1=0 days to 7=All 30 days (coded 1=0 and 2–7=1)”
Physically attacked	“During the past 12 months, how many times were you physically attacked?”	“1=0 times to 8=12 or more times (coded 1=0 and 2–8=1)”
Physical fighting	“During the past 12 months, how many times were you in a physical fight?”	“1=0 times to 8=12 or more times (coded 1=0 and 2–8=1)”
**Oral and hand hygiene**		
Brushing teeth (≤1 time/day)	“During the past 30 days, how many times per day did you usually clean or brush your teeth?”	“1=never to 6=4 or more times a day (coded 1-3=1 and 4-6=0)”
Hand washing before eating	“During the past 30 days, how often did you wash your hands before eating?”	“1=never to 5=always (coded 1–4=1 and 5=0)”
Hand washing with soap	“During the past 30 days, how often did you use soap when washing your hands?”	“1=never to 5=always (coded 1–4=1 and 5=0)”
Hand washing after toilet	“During the past 30 days, how often did you wash your hands after using the toilet or latrine?”	“1=never to 5=always (coded 1–4=1 and 5=0)”
**Poor mental health indicators**		
No close friends	“How many close friends do you have?”	“1 = 0 to 4 = 3 or more (coded 1+=0, 0=1)”
Loneliness	“During the past 12 months, how often have you felt lonely?”	“1=never to 5=always (coded 1–3=0 and 4–5=1)”
Worry-induced sleep disturbance	“During the past 12 months, how often have you been so worried about something that you could not sleep at night?”	“1=never to 5=always (coded 1–3=0 and 4–5=1)”
Suicidal ideation	“During the past 12 months, did you ever seriously consider attempting suicide?”	“Yes, No”
Suicide plan	“During the past 12 months, did you make a plan about how you would attempt suicide?”	“Yes, No”
**Protective factors**		
Peer support	“During the past 30 days, how often were most of the students in your school kind and helpful?”	“1=never to 5=always (coded 1–3=0 and 4–5=1)”
School truancy	“During the past 30 days, on how many days did you miss classes or school without permission?”	“1=0 days to 5=10 or more days (coded 1=0 and 2-5=1)”
Parental supervision	“During the past 30 days, how often did your parents or guardians check to see if your homework was done?”	“1=never to 5=always (coded 1–3=0 and 4–5=1)”
Parental connectedness	“During the past 30 days, how often did your parents or guardians understand your problems and worries?”	“1=never to 5=always (coded 1–3=0 and 4–5=1)”
Parental bonding	“During the past 30 days, how often did your parents or guardians really know what you were doing with your free time?”	“1=never to 5=always (coded 1–3=0 and 4–5=1)”

### Covariates

We categorized age into three groups (≤ 11–13, 14–15, and ≥ 16 years), experience of hunger (as a proxy for socioeconomic status) into three groups (never, rarely or sometimes, and most of the time or always) and study year into three groups (2005, 2010, and 2016), with the first value being the reference category, respectively.

### Data analysis

Statistical analyses were conducted using “STATA software version 15.0 (Stata Corporation, College Station, Texas, USA)”. Data were weighted for non-response and probability selection [3]. In order to test for differences in proportion Pearson Chi-square tests were utilized. Logistic regression analyses were applied to estimate each health indicator outcome adjusted by age group, socioeconomic status (experience of hunger) and study year for boys and girls, separately. In order to account for the sample weight and the multi-stage sampling design, Taylor linearization methods were applied. Results from the logistic regression analyses are shown as odds ratios (ORs) with 95% confidence intervals (CIs). Missing values were excluded from the analysis. P < 0.05 was considered significant.

## Results

### Description of the study sample

Across the 2005, 2010, and 2016 UAE GSHS the overall sample consisted of 24,220 school-going adolescents, 52.2% females and 47.8% males (median age = 14 year, interquartile range = 2 years). The number of older adolescents increased across the three different assessment years (*P* < 0.001) (see Table 2).

**Table 2:** Sample characteristics of school adolescents: 2005, 2010 and 2016 surveys in UAE

Variable	2005 (*N* = 15,790)	2010 (*N* = 02,581)	2016 (*N* = 05,849)	Total (*N* = 24,220)
	N (%)	N (%)	N (%)	N (%)
Gender				
Male Female Missing	7741 (50.0) 7893 (50.0) 156 (0.9)	1079 (42.1) 1483 (57.9) 19 (0.8)	2763 (49.7) 3041 (50.3) 45 (0.7)	11,583 (47.8) 12,417 (52.2) 220 (0.8)
Age in years				
11 or younger 12 13 14 15 16 years or older Missing	404 (2.6) 2150 (13.1) 3630 (22.3) 3827 (23.6) 3212 (21.2) 2373 (17.2) 194 (1.1)	9 (0.4) 123 (4.3) 669 (23.1) 846 (31.8) 664 (29.2) 259 (11.2) 11 (0.4)	41 (0.7) 281 (4.7) 911 (13.9) 1126 (19.8) 1153 (19.8) 1314 (41.1) 23 (0.4)	454 (1.2) 2554 (7.1) 5210 (18.7) 5799 (24.0) 5029 (22.6) 4946 (26.4) 228 (0.6)
Grade				
7 8 9 10 and other Missing	4215 (26.9) 4064 (25.4) 3851 (24.2) 3431 (23.5) 228 (1.4)	945 (33.7) 939 (33.9) 677 (32.4) 0 20 (0.7)	244 (4.6) 1215 (16.8) 1156 (22.2) 3118 (35.9) 116 (1.9)	5404 (18.7) 6219 (23.7) 5684 (25.4) 6519 (32.2) 364 (1.5)

### Health indicator outcomes

#### Overweight and poor diet

Among students, 21.2% of males and 21.7% of females were overweight or obese in 2005, while this significantly increased among boys in 2010 (43.7%) and 2016 (42.1%) as well as significantly increased but to a lesser extent among girls than boys in 2010 (36.0%) and 2016 (35.6%). Likewise, the prevalence of obesity significantly increased over time among boys but not among girls. More than two in three male students (68.7%) and 75.2% female students had less than two servings of fruits per day in 2005, while these prevalences significantly decreased between both sexes in 2016. Inadequate vegetable intake significantly reduced between both sexes from 2005 to 2016. Among girls, the proportion of experiencing hunger reduced from 2005 to 2010 but stayed unchanged from 2005 to 2016, while hunger experiences reduced among boys from 2005 to 2016.

#### Physical activity and sedentary behaviour

The prevalence of inadequate physical activity did not change among boys but increased among girls over time, and the proportion of sedentary behabiour significantly increased from 2005 to 2016 among both boys and girls.

#### Tobacco use

The prevalence of current tobacco use increased among both boys and girls over time but this was not statistically significant.

#### Injury and violence

Having been physically attacked and involved in physical fighting significantly decreased among boys and physical assault decreased among girls from 2005 to 2016, while the prevalence of injury increased significantly in both sexes from 2005 to 2016. Bullying victimization increased among both boys and girls from 2005 to 2016.

#### Oral and hand hygiene

The prevalence of inadequate oral hygiene (tooth brushing) was 48.6% among male and 37.9% among female students in 2005, while this remained unchanged among boys a significant reduction was found among girls in 2010 and 2016. Not always washing hands prior to eating significantly increased among both sexes from 2005 to 2010 and 2016, while the other two poor hand washing indicators (not always washing hands after toilet use and with soap) did not significantly change over time among both boys and girls.

#### Poor mental health

Loneliness decreased among both boys and girls from 2005 to 2016, while there was no significant change for the remaining four poor mental health indications (worry-induced sleep disturbance, having no close friends, suicide plan and suicidal ideation).

#### Protective factors

 Among both girls and boys, peer support did not change from 2005 to 2016, and truancy did not change among boys but increased among girls over time. Among the three parental support indicators (bonding, connectedness and supervision), all remained unchanged over time except for a decrease in parental supervision among boys and girls (see Tables 3 and 4).

**Table 3:** Health risk indicators in 2005, 2010 and 2016 among male school adolescents, UAE

*Variable*	2005	2010	2016	Change over time compared to 2005	
	N (%)	N (%)	N (%)	2010 Adjusted^a^ OR (95% CI)	2016 Adjusted^a^ OR (95% CI)
***Body weight and dietary behaviour***					
Overweight or obesity	1510 (21.2)	421 (43.7)	1074 (42.1)	2.93 (2.45, 3.50)***	2.82 (2.41, 3.32)***
Obesity	928 (13.2)	186 (19.8)	556 (21.3)	1.69 (1.37, 2.10)***	1.77 (1.46, 2.14)***
Fruits < 2 day	5275 (68.7)	753 (70.6)	1695 (61.6)	1.07 (0.93, 1.23)	0.63 (0.53, 0.76)***
Vegetable < 3 day	6205 (81.0)	853 (79.9)	2159 (78.4)	0.92 (0.79, 1.07)	0.77 (0.66, 0.89)***
Went hungry (mostly/always)	815 (10.0)	59 (6.2)	241 (7.9)	0.60 (0.43, 0.85)**	0.70 (0.55, 0.90)**
***Physical activity and sedentary behaviour***					
Inadequate physical activity	5768 (77.1)	797 (77.5)	2150 (79.7)	1.04 (0.85, 1.27)	1.10 (0.94, 1.29)
Leisure-time sedentary behaviour	2814 (38.0)	475 (45.0)	1322 (51.1)	1.27 (1.04, 1.55)*	1.53 (1.30, 1.80)***
***Current tobacco use***	1394 (13.2)	251 (19.8)	705 (21.3)	1.24 (0.98, 1.56)	1.10 (0.88, 1.36)
***Injury and violence***					
Any serious injury (past year)	2243 (38.4)	481 (51.8)	1219 (51.0)	1.81 (1.57, 2.09)***	1.72 (1.50, 1.97)***
Bullied (past month)	1714 (24.5)	260 (25.9)	810 (29.9)	1.14 (0.95, 1.36)	1.41 (1.20, 1.67)***
In physical fight (past year)	4329 (56.9)	646 (60.9)	1403 (50.8)	1.20 (1.01, 1.44)*	0.84 (0.71, 0.99)*
Physically attacked (past year)	3100 (40.8)	448 (42.0)	916 (32.8)	1.15 (0.97, 1.37)	0.79 (0.69, 0.91)**
***Oral and hand hygiene***					
Brushing teeth (≤ once/day)	3665 (48.6)	498 (46.8)	1270 (46.1)	0.91 (0.73. 1.12)	0.83 (0.68, 1.02)
Wash hands before eating (not always)	2210 (29.6)	413 (38.7)	1081 (41.6)	1.53 (1.19, 1.97)***	1.67 (1.34, 2.08)***
Wash hands after toilet/ latrine use (not always)	1271 (17.1)	203 (19.3)	554 (19.2)	1.24 (1.02, 1.51)*	1.13 (0.90, 1.43)
Wash hands with soap (not always)	2580 (34.9)	363 (34.0)	912 (33.1)	0.97 (0.82, 1.14)	0.92 (0.78, 1.10)
***Poor mental health***					
Having no close friends	478 (6.6)	74 (7.2)	205 (7.0)	1.16 (0.86, 1.56)	1.14 (0.84, 1.55)
Loneliness (past year)	967 (13.1)	166 (15.9)	329 (11.6)	1.32 (1.03, 1.69)*	0.76 (0.64, 0.90)**
Worry-induced sleep disturbance (past year)	792 (10.6)	140 (13.0)	331 (11.9)	1.35 (1.05, 1.73)*	1.00 (0.85, 1.19)
Suicidal ideation (past year)	945 (13.4)	147 (14.7)	199 (12.5)	0.93 (0.70, 1.22)	0.95 (0.69, 1.30)
Suicide plan (past year)	692 (10.3)	145 (14.2)	154 (9.5)	1.29 (1.03, 1.57)*	0.93 (0.69, 1.22)
***Protective factors***					
Truancy (past month)	2461 (34.0)	387 (38.3)	1108 (40.2)	1.19 (0.93, 1.53)	1.10 (0.87, 1.39)
Peer support (mostly/always)	4167 (55.6)	590 (56.7)	1521 (56.9)	1.00 (0.82, 1.23)	1.03 (0.86, 1.24)
Parents/guardians supervision (mostly/always)	4055 (54.8)	522 (52.3)	1248 (44.7)	0.90 (0.74, 1.10)	0.72 (0.60, 0.85)***
Parents/guardians connectedness (mostly/always)	3634 (50.1)	465 (45.0)	1162 (45.2)	0.79 (0.67, 0.94)**	0.88 (0.76, 1.02)
Parents or guardians bonding (mostly/always)	3935 (52.9)	478 (46.1)	1319 (49.9)	0.75 (0.62, 0.91)**	1.03 (0.87, 1.21)

*OR *Odds Ratio, *CI *Concidence Interval

^a^Adjusted for age group, experiences of hunger (proxy measure for socioeconomic status) (except for hungry as outcome) and study year; ***P < 0.001; **P < 0.01; *P < 0.05;

**Table 4:** Health risk indicators in 2005, 2010 and 2016 among female school adolescents, UAE

***Variable***	2005	2010	2016	Change over time compared to 2005	
	N (%)	N (%)	N (%)	2010 Adjusted^a^ OR (95% CI)	2016 Adjusted^a^ OR (95% CI)
***Body weight and dietary behaviour***					
Overweight or obesity	1589 (21.7)	484 (36.0)	1013 (35.6)	2.05 (1.69, 2.48)***	2.09 (1.77, 2.46)***
Obesity	782 (11.0)	177 (12.4)	383 (13.0)	1.20 (0.93, 1.56)	1.16 (0.98, 1.39)
Fruits <2 day	5798 (75.2)	1110 (76.2)	2138 (68.6)	1.05 (0.85, 1.29)	0.62 (0.49, 0.80)***
Vegetable <3 day	6602 (84.7)	1232 (84.8)	2459 (79.5)	0.96 (0.80, 1.17)	0.63 (0.52, 0.78)***
Went hungry (mostly/always)	672 (8.9)	72 (5.0)	332 (9.9)	0.56 (0.38, 0.85)**	1.00 (0.77, 1.30)
***Physical activity and sedentary behaviour***					
Inadequate physical activity	6513 (83.8)	1250 (86.7)	2660 (88.6)	1.26 (0.99, 1.60)	1.37 (1.14, 1.66)***
Leisure-time sedentary behaviour	3019 (39.6)	790 (56.0)	1974 (66.7)	1.90 (1.53, 2.30)***	2.63 (2.21, 3.12)***
***Current tobacco use***	453 (5.7)	121 (9.3)	293 (10.4)	1.81 (1.15, 2.84)*	1.43 (0.98, 2.10)
***Injury and violence***					
Any serious injury (past year)	1243 (19.0)	464 (35.1)	940 (34.5)	2.48 (2.07, 2.97)***	2.22 (1.84, 2.67)***
Bullied (past month)	1292 (17.2)	297 (20.7)	605 (20.5)	1.37 (1.11, 1.69)**	1.27 (1.05, 1.53)*
In physical fight (past year)	2329 (29.5)	539 (36.5)	806 (26.5)	1.48 (1.26, 1.74)***	0.91 (0.73, 1.13)
Physically attacked (past year)	1838 (23.0)	411 (28.5)	555 (18.2)	1.53 (1.24, 1.88)**	0.79 (0.64, 0.97)*
***Oral and hand hygiene***					
Brushing teeth (≤once/day)	2801 (37.9)	450 (31.4)	870 (28.3)	0.76 (0.63, 0.93)**	0.60 (0.48, 0.75)***
Wash hands before eating (not always)	2385 (31.9)	603 (40.5)	1431 (49.0)	1.44 (1.16, 1.78)***	2.03 (1.67, 2.47)***
Wash hands after toilet/ latrine use (not always)	1149 (15.3)	236 (15.6)	549 (17.7)	1.10 (0.89, 1.36)	1.15 (0.98, 1.35)
Wash hands with soap (not always)	2175 (28.3)	496 (32.7)	912 (30.6)	1.27 (0.99, 1.62)	1.19 (0.97, 1.47)
***Poor mental health***					
Having no close friends	489 (6.2)	76 (5.3)	204 (6.6)	0.89 (0.68, 1.17)	1.01 (0.81, 1.26)
Loneliness (past year)	1368 (17.7)	260 (17.9)	485 (16.0)	1.14 (0.93, 1.39)	0.72 (0.59, 0.88)**
Worry-induced sleep disturbance (past year)	1360 (18.1)	270 (19.4)	628 (20.5)	1.20 (0.97, 1.49)	0.95 (0.79, 1.14)
Suicidal ideation (past year)	982 (12.5)	240 (17.5)	289 (15.2)	1.13 (0.85, 1.51)	0.98 (0.76, 1.06)
Suicide plan (past year)	717 (9.2)	234 (16.9)	241 (12.4)	1.65 (1.32, 2.05)***	1.25 (0.97, 1.64)
***Protective factors***					
Truancy (past month)	2089 (28.3)	526 (37.2)	1335 (41.4)	1.63 (1.32, 2.01)***	1.41 (1.03, 1.92)*
Peer support (mostly/always)	5488 (71.0)	1036 (72.0)	2104 (68.0)	1.00 (0.83, 1.21)	0.87 (0.70, 1.07)
Parents/guardians supervision (mostly/always)	3646 (47.9)	588 (41.6)	1221 (39.0)	0.75 (0.59, 0.95)*	0.78 (0.63, 0.98)*
Parents/guardians connectedness (mostly/always)	3960 (51.2)	691 (47.1)	1428 (47.7)	0.80 (0.67, 0.94)**	0.96 (0.82, 1.13)
Parents or guardians bonding (mostly/always)	4477 (58.3)	756 (51.6)	1676 (56.6)	0.72 (0.62, 0.84)***	1.06 (0.87, 1.29)

*OR *Odds Ratio, *CI *Concidence Interval

^a^Adjusted for age group, experiences of hunger (proxy measure for socioeconomic status) (except for hungry as outcome) and study year; ***P<0.001; **P<0.01; *P<0.05;

## Discussion

The study found across the 2005, 2010 and 2016 GSHS in UAE a significant reduction of being physically attacked, inadequate fruit intake, inadequate vegetable consumption, and loneliness among both boys and girls, and among girls only poor oral hygiene (< 2 times tooth brushing/day) and among boys only, experiencing hunger and in physical fight. Among both boys and girls significant rises were identified in the prevalence of bullying victimization, overweight or obesity, leisure-time sedentary behaviour, injury and not always washing hands prior to eating, and among boys only obesity and among girls only inadequate physical activity, and school truancy.

In 2004, the national health promoting school network was implemented in UAE, including the promotion of healthy behaviour (diet, physical activity, safety, mental, emotional and social health, comprehensive screening [21]. In a recent study among adolescents in Dubai, UAE, more than one in four had limited health literacy, calling for health literacy training among UAE adolescents [22]. A strengthening of the health promotion school activities is indicated in order to improve on some of health indicators.

The study showed a stark increase of overweight and obesity in this study from 2005 to 2010 and 2016, in both boys and girls and even a greater increase among boys than girls did. Previous studies, (e.g. [10]) have reported high rates of overweight and obesity among adolescents in UAE, including a steady rise in obesity, especially in boys [23]. These findings seems to be consistent with global increases in the prevalence of obesity among adolescents from 1975 to 2016 [24]. In the 2005 UAE GSHS insufficient fruit and vegetable consumption was high and further increased to 2016. Similar increases in inadequate fruit and vegetable intake were also shown in a trend study in Oman [6] and other countries in the Arab region [25]. The prevalence of experiencing hunger was low and significantly reduced among boys but not girls from 2005 to 2016.

Violence-related events (in a physical fight and physical assault) reduced in the present study over time. Similar results were found in four other research studies [4, 26–28], while in Oman [6], the Philippines [5] and Venezuela [29] one or more types of interpersonal violence increased. Several local studies among adolescents in UAE have stressed the importance of interpersonal violence [8, 9] and this study found an increase in bullying victimization among boys and girls over time. This result may call for anti-bullying programmes among school adolescents in UAE. However, among both boys and girls the prevalence of annual injury significantly increased, which is consistent with the trend study in the Philippines [5]. On the other hand, the injury prevalence among adolescents in Morocco declined [30], and no significant trend differences were identified in Oman [6]. The large increase in the occurrence of injuries calls for school safety promotion and injury prevention among adolescents in UAE.

Physical inactivity increased among female students in this study. Henry et al. [31] concluded from a study among female adolescents in the UAE that the physical activity was very low, attributing this to weather and cultural restrictions as well as unconducive community attitudes [31]. Leisure- time sedentary behaviour increased significantly in this study to 51.1% in boys and 66.7% in girls, which is much higher than the global average in school-going adolescents (26.4%) [32] and the highest among 10 Eastern Mediterranean countries [33]. Since in this study, leisure-time sedentary behaviour was assessed with a composite measure ”sitting and watching television, playing computer games, talking with friends, or doing other sitting activities, such as studying or using any electronic devices like IPads” [3], we are not able to identify if a particular type of sedentary behaviour increased more than another type. Some studies, e.g., in the US, showed an increase of the use of recreational screen-based devices, such as electronic entertainment and computer use, among adolescents during the first decade of the 21st century [34], which may be applicable to the UAE too.

The proportion of inadequate tooth brushing (< twice/day) was high across the three UAE GSHS (> 46% in boys and > 30% in girls), significantly higher than among adolescents in Southeast Asia (22.4%) [35]. In a survey among private school adolescent students in Abu Dhabi, Dubai, 63.6% had sub-optional oral hygiene practices [36], and in a sample of adolescent school children in Sharjah, UAE, 19.8% of Emirati and 40.3% other Arabs engaged in inadequate tooth brushing (< 2 times/day) [37], indicating the importance of improving oral health hygiene in UAE. Poor hand washing before eating increased in both sexes in this study, which was similar in the Oman trend study [6], while poor hand hygiene decreased among adolescents in the Philippines [5]. In a study among primary school students in Sharjah, UAE, 27% did not always wash hands before eating and 31% did not always wash hands after toilet use [38], and in Al Anin, UAE, among 15 to 55 year-olds from the community “30% did not always wash their hands before and after eating and 20% did not always wash their hands after using toilets.” [39]. All the more, an improvement of hand hygiene behaviour among adolescents in UAE is indicated.

The prevalence of current tobacco use increased among both boys and girls over time but this was not statistically significant, and concur with previous investigations in the UAE [13]. On the other hand the prevalence of current tobacco use from the UAE Global Youth Tobacco Survey (GYTS) in 2005 (19.5%) decreased to 12.2% in 2013 [40, 41]. In terms of four indicators of mental health (suicide plan, suicidal ideation, worry-induced sleep disturbance, and having no close friends), the study did not find significant changes over time, except for a decrease in loneliness in both sexes. While the prevalence of loneliness increased among both boys and girls over time in the Philippines trend study [5]. As shown in some previous studies among adolescents in UAE [14, 15], mental morbidity in the form of depressive and anxiety-related symptoms has been shown as to be a significant burden.

Consistent with previous studies [4–6], this survey found mixed results on protective factors, parental support indicators did not change except for a decrease of one parental indicator (parental supervision) among both girls and boys, peer support did not change, and school truancy increased among girls. For example, in the New Zealand trend study positive school and family connections became better over time [4], in the Oman trend study peer support increased over time [5], and in the Philippines trend study protective factors remained unchanged over time [6].

The present research findings may contribute to better targeting of specific health indicators among adolescents in health promotion activities in UAE. For example, school-based interventions can be effective in reducing excessive weight gain and in promotion of physical activity and fitness [42, 43]. After-school programmes can improve physical activity levels [44]. Dietary behaviours may be improved by implementing specific school food environment policies, such as the direct provision of healthy beverages and foods [45]. In the prevention of bullying and smoking different types of whole-school health interventions have shown to be effective. [46] Poor mental health (anxiety and depressive symptoms) among adolescents may be decreased by universal resilience-focused interventions (especially cognitive-behavioural therapy) [47]. Increased implementation of multi-level (training, funding and policy) interventions have shown to reduce absenteeism from school, respiratory infections and diarrhoea [48].

### Limitations of the study

Secondary education enrolment ratio was 95.3% in UAE in 2016 [49], meaning that out-off school adolescents were excluded in this UAE GSHS. A few study variables (such as alcohol use, drug use and sexual behaviour) were excluded in the present analysis, since they had not been measured in all three of the UAE GSHS. Further study limitations include the cross-sectional study design and the self-report of the data, in particular height and body weight. Several studies [50, 51] comparing self-report and measured height and weight among adolescents, conclude that self-reported BMI may be used as a valid tool to estimate BMI overweight/obesity in epidemiological studies and that self-reported BMI may be an underestimate. Further, it has been shown in previous research that anonymous self-report questionnaires may generate more accurate data on sensitive variables compared to other methods among adolescents [52, 53].

## Conclusions

In three nationally representative surveys of in-school adolescents over a period of 11 years in the UAE, a significant reduction of being physically attacked, inadequate fruit intake, inadequate vegetable consumption, and loneliness were found among both boys and girls, while among girls only poor oral hygiene (< 2 times tooth brushing/day) and among boys only, experiencing hunger and in physical fight declined. Significant rises were identified among both sexes in the prevalence of bullying victimization, overweight or obesity, leisure-time sedentary behaviour, injury and not always washing hands prior to eating, and among boys only obesity and among girls only inadequate physical activity, and school truancy. Several poor health indicators declined but even more increased over three cross-sectional surveys from 2005 to 2016 emphasizing the need for enhanced health promotion activities in this adolescent school population.

## Abbreviations

GSHS: Global School-Based Student Health Survey
STATA: Statistics and data
UAE: United Arab Emirates

## Availability of data and materials

The data for the current study are publicly available at the World Health Organization NCD Microdata Repository (URL: https://extranet.who.int/ncdsmicrodata/index.php/catalog).
